# Adoption of ICT4D and its determinants: A systematic review and meta-analysis

**DOI:** 10.1016/j.heliyon.2024.e30210

**Published:** 2024-04-25

**Authors:** Rico Amoussouhoui, Aminou Arouna, Sacha Ruzzante, Jan Banout

**Affiliations:** a Department of Sustainable Technologies, Faculty of Tropical AgriSciences, Czech University of Life Science in Prague, Kamýcká 129, 165 00, Prague, Czech Republic; b Africa Rice Center (AfricaRice), 01 BP 2551 Bouake 01, Bouake, Cote d’Ivoire; c Department of Civil Engineering, University of Victoria, 3800 Finnerty Rd, Victoria, BC, V8P 3E6, Canada

**Keywords:** Agriculture 4.0, Digital agricultural technologies, Meta-analysis, Adoption

## Abstract

Various Digital Agricultural Technologies (DAT) have been developed and implemented around the world. This study aims to estimate the overall adoption rate and identify the determinant factors for a better adoption perspective after decades of innovation and dissemination. A systematic review was conducted on published studies that reported adoption rates and determinant factors using the Preferred Reporting Items for Systematic Reviews and Meta-Analyses (PRISMA) protocol. We used meta-regression and the partial correlation coefficient to estimate the effect size and establish the correlation between socioeconomic characteristics and the adoption of various technologies reported. Fifty-two studies with 32400 participants met the selection criteria and were included in the study. The results revealed an overall pooled adoption rate of 39 %, with the highest adoption rates in developing countries in Africa and South America. Socioeconomic factors such as age, education, gender, and income were found to be the main determinants and should be considered when designing technology for sustainable adoption. The study also found that young farmers were more susceptible to adoption. Moreover, farmers with higher income levels and educational attainment are more likely to use technology linked to agricultural production, market access, and digital advising, implying that high-income farmers with more education are more tech-savvy. However, this does not exclude low-income and low-educated farmers from adopting the technologies, as many models and strategies with socioeconomic considerations were developed. It is one of the reasons behind the underlying enthusiasm for digital agricultural adoption in low and middle-income countries.

## 1 Introduction

The rapid growth of the world population, climate change, and its negative impact on the environment and food security have been significant challenges faced by global agriculture in the last 50 years [1,2]. Information and Communication Technology (ICTs) has provided new opportunities and alternatives for economic development in various sectors, including agriculture, by allowing farmers to reduce constraints and improve their value chain [3,4]. In recent decades, Digital Agricultural Technologies (DAT) have offered diverse opportunities to address challenges and enhance farmer resilience [2,5], including smart devices, intelligent systems supported by interconnected networks, and cloud computing [6]. This provides small, medium, and industrialized farm holders with an intelligent solution to transform conventional agricultural systems [7,8]. These innovations are expected to lead to the fourth agricultural revolution (Agriculture 4.0), which aims to improve agricultural development, offer better ecosystem services, and establish a future for reliable and sustainable agriculture [9,10]. Even though there is still an open discussion in the literature regarding the meaning of digital technologies, this study focuses on the most common definition of DAT, which includes smart devices, big data, and precision agricultural technologies [2,11]. It implies technologies such as global positioning systems, remote sensing, smart devices, robotics, and cloud-based decision support tool software. DAT positively impacts agricultural development by increasing productivity, resource efficiency, and climate change resilience [12]. In addition, it can enhance the whole value chain productivity and help manage unpredicted situations such as the COVID-19 pandemic [13]. Due to their levels of education, better access to credit, and higher purchasing power, high-income countries (HICs) are hypothetically more likely to use digital technologies. In contrast, many digital agricultural innovations are being developed and introduced in low- and middle-income countries (LMICs). Still, due to weak infrastructure, limited digital literacy, poverty, and other reasons, few farmers have adopted DAT thus far. This is one of the reasons why the literature is more focused on LMICs regarding DAT adaptation [13]. Indeed, adopting DAT in LMICs is a new, promising perspective regarding its economic, social, and environmental impact [13,14]. Alternatively, the adoption of communication tools for agricultural purposes is growing [15] through the use of communication channels such as WhatsApp, Twitter, Zoom, and YouTube to share information [16], as well as Android software developed for this purpose. The growth of DAT is considered a pillar for the Agriculture 4.0 revolution, given the expected impact, especially in developing countries where agriculture is the pith of economic development [[17], [18], [19]]. DAT does not imply necessary or only precision machines but tools such as digital devices, applications, and other digital platforms accessible through smartphones for agricultural purposes [20].

Some initiatives have emerged in Africa, such as the RiceAdvice technology developed for rice farming [21]. In East Africa, where the digital agricultural initiative started in Africa, approximately 60 % of farmers use digital technology [22]. The former Technical Center of Agricultural and Rural Cooperation (CTA) estimated that approximately 10 % of farmers and pastoralists in sub-Saharan Africa use some digital service [20]. The literature has shown that DAT can positively impact yields, productivity, food security, and rural incomes [21,23,24]. However, despite the numerous promised advantages and interests, there are challenges and risks linked to accessibility, such as the cost of the technologies, limits when considering local ecological knowledge, and the complexity of the technologies [25,26]. These challenges explain the low adoption of precision agricultural technologies registered in the past years [27,28], especially in developing countries. Among these challenges, literature has focused more on accessibility, a preliminary step to adoption. The expected impact of DAT can be achieved if most farmers adopt them.

We found similar studies in the literature, but they show some limitations and do not quantitatively focus on the adoption rate and determinants. Through a literature review performed only on Web of Science, Shang et al. [29] worked on adopting DAT but did not use the Cochrane guidelines or PRISMA protocol. Benyam et al. [25] used Scopus, Web of Science, ScienceDirect, Connected Papers, Google Scholar, and Google to analyze a global trend, adoption opportunities, and barriers of DAT regarding food loss and waste prevention and reduction. Abbasi et al. [30] accessed the digitization of the agricultural industry, and Porciello et al. [31] studied digital agriculture services in low and middle-income countries. Although Benyam et al. [25], Abbasi et al. [30], and Porciello et al. [31] used a systematic review approach, a meta-analysis of the adoption rate and determinants was not done.

Nevertheless, there is a lack of information on the global adoption of DAT and the determinant factors. The study should determine the adoption level globally for each technology type and highlight the socioeconomic determinants that drive adoption. First, the global level of adoption would tell us how the technologies are being adopted and at which level. Second, what are the socioeconomic factors that drive adoption? This information would enable us to determine which type of technology has more potential in the future and which socioeconomic characteristics to consider when designing a tailor-made technology that is more likely to be adopted. To our knowledge, no study has provided extensive information on this matter that would guide future efforts and investments to target better and improve accessibility and adoption of DAT. To fill this literature gap, this study aims to conduct a systematic review and a meta-analysis of the published research papers related to the adoption of DAT to answer the following research questions.

(i)To what extent do farmers adopt DAT? What is the overall adoption rate of DAT worldwide?

The answer to these questions would provide a global view of DAT's adoption rate and technology type. This information will help technology developers, policymakers, and development partners develop better strategies and policies.

(ii)Does the adoption rate vary by socioeconomic characteristics such as age, education, gender, income, and publication year?

We first search for a correlation between socioeconomic factors and the adoption rate to understand and identify the factors that drive adoption. We also search for a correlation between publication year and the adoption rate to appreciate the trend of research related to adoption over time.

(iii)How do small studies affect the estimated overall adoption rate?

This research question aims to evaluate the effect of a small study on the overall adoption rate to check if it affects the overall result and controls the bias.

(iv)What is the correlation between the effect size (ES) of socioeconomic characteristics and the characteristics of DAT?

A better understanding of these issues may help promote and ensure farmers' sustainable adoption of DAT. This is a first step toward agricultural sustainability and economic growth. This study proposes a critical and comprehensive review using empirical studies. Note that the uniqueness and novelty of the study can be summarized in three lines. To the best of our knowledge, this is the first study to estimate the global adoption rate and effect size of DAT and the determinants of their adoption, using a systematic review, a meta-analysis, and the partial correlation coefficient (PCC) approach. Even though the literature has broadly addressed studies on the adoption of specific digital agricultural technology, to ensure a sustainable adoption of the technology, it is essential to analyze the adopter's behavior based on their socioeconomic characteristics and explore how the individual socioeconomic characteristics affect the adoption of a technology or another. This is an important outcome of designing more suitable technologies and developing an adequate adoption approach. Based on each continent's socioeconomic realities, our study provides the first in-depth analysis of the adoption of DAT and their potential contribution to development. We provided a quantitative overview of the adoption of DAT, categorized the different types of technologies, and identified the key factors that drive their adoption. Above all, our research's theoretical contribution to literature is twofold. This research contributes to understanding the adoption of ICT4D and the scientific knowledge in systematic review applied to adopting new technologies.

In addition, our study collects and analyzes quantitative data on technology adoption and its determinants and conducts a systematic review using the well-known PRISMA protocol. Furthermore, we collect and categorize various DATs worldwide. This distinguishes and differentiates our paper, which provides policymakers, technology developers, and development institutions with more quantitative information.

## 2 Materials and methods

### 2.1 Article identification strategy

**Fig. 1:**
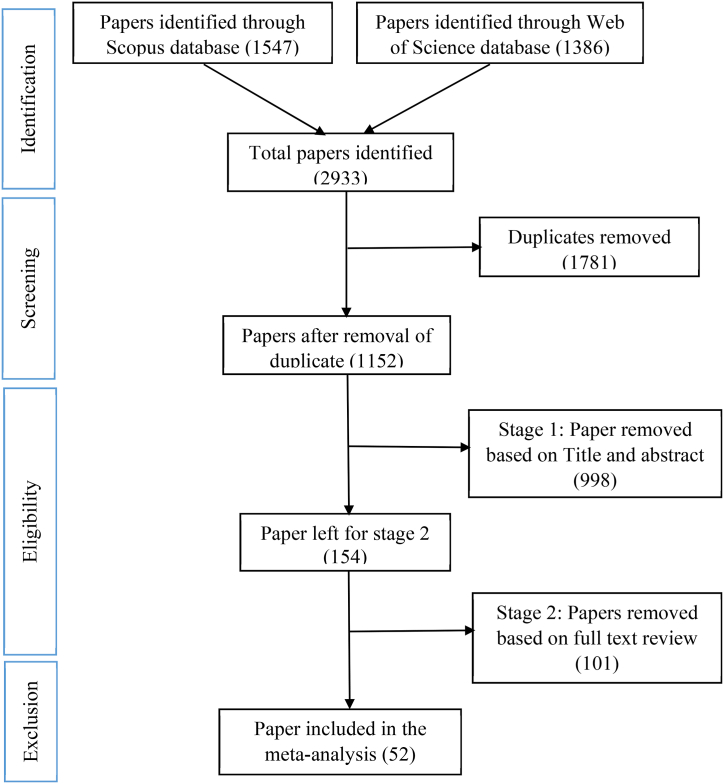
PRISMA diagram of the paper collection.

We performed a systematic review following the Cochrane guidelines [32] and the Preferred Reporting Items for Systematic Reviews and Meta-Analyses (PRISMA) protocol [33]. The aim was to identify quality research papers on adopting DAT. We were interested in evaluating how farmers adopt digital agricultural technology and digital technology/devices not designed for agriculture but used for agricultural purposes. Based on a recent study examining the relevant and suitable academic search system for systematic review [34]. The Web of Science (WoS) and Scopus search engine databases were used as the sources of information. Only peer-reviewed articles were selected. The search focused on the last 20 years to have the maximum number of published papers fitting the criteria. The keywords used to identify the papers were: Adoption of digital agricultural technology; Adoption of digital farming technology; DAT; Agriculture digital technology adoption; ICT adoption of agriculture; Determinant adoption of ICT agriculture; Determinant adoption of digital technologies agricultural; Determinant adoption of digital farming technologies; and Agriculture 4.0 adoption (Supplemental S1). Fig. 1 presents the PRISMA flowchart describing the data collection process following the systematic review protocol. The final sample of 52 studies (focusing on the adoption and determinants of DAT) with 67 adoption rates were recorded when including the studies with more than one technology.

### 2.2 Article eligibility

The review results highly depend on the quality of the papers used for the meta-analysis. To ensure the quality of the selection, we used the following strategy. First, we reviewed the titles and abstracts of the papers obtained using the keywords in the search engines. If the abstract was not explicit enough, we read through all the content. We defined inclusion and exclusion criteria (Table 1) for selecting relevant articles that we used for the meta-analysis.

**Table 1:** Article selection criteria.

Criteria	Inclusion	Exclusion
Language	English	Other languages
Peer-review and publication status	Fully peer-reviewed and published	Not fully peer-reviewed or unpublished
Topic	Related to the adoption of DAT	ICT tool adoption by farmers, Agricultural extension in general, Private agricultural extension in general
Evaluation of determinants of DAT adoption	Yes	–
Methodology	Quantitative data and econometric methods	–
Sample size	Random samples with a minimum size of 30	–
Review type	–	Qualitative review on agricultural extension

### 2.3 Article classification

DAT involves using different digital technologies for various reasons and uses. We first adopted the classification by Abbie [35] (Table 2), which provides a classification for the LMIC.

**Table 2:** Classification of digital agricultural technologies by abbie.

Access to services		Access to markets		Access to assets
Digital advisory	Agri digital financial services	Digital procurement	Agri e-commerce	Smart farming
Agri VAS	Credit and loans	Digital records	Inputs	Smart shared assets
Smart advisory	Input financing	Digital records with payments	Outputs	Equipment monitoring
Weather information	Credit scoring	Digital records with traceability	Inputs and Outputs	Livestock and fishery management
Pest and disease management	Crowdfunding	Digital records with payments and traceability		
Product verification	Insurance			
Record keeping	Digital agri wallet			
	Savings			
	Accountability tool			

However, this classification does not necessarily consider high-income countries. It does not fit with all types of technologies registered, for example, when a smartphone or a phone is used for agricultural purposes. Therefore, studies were categorized into three groups considering the nature of the technology and its location. The first group is the technology field, which refers to the field in which/for which the technology is used, with two options: Crop production and livestock. The second group is the type of technology, with two options: Digital technology and Precision agriculture. The third group is the continent.

### 2.4 Selection process and extraction

The selection process was performed based on the criteria, two independent author reviews, and discussions with a third author reviewer in case of discordance between the two independent reviewers. The selection process started with an initial screening of the title and the abstract of the study papers collected from the database engine using the above keywords. Afterward, we removed the unsuitable papers, and in case there was uncertainty, the pair of reviewers went through the main text to see whether the article met the criteria [36]. The full-text papers were uploaded to a reference manager, which helped remove the duplicate articles using the DOI. We also used Microsoft Excel to report each selection phase and organized the selected papers and the data collected. We collected and extracted data from the selected papers for the meta-analysis. We extracted two types of data.

• General data: title, authors, study area, and publication year.
• Specific data: sample size, number of adopters, type of DAT, adoption rate, and coefficient and standard error of socioeconomic variables: age, gender, education, farming size, and income. Note the variable gender is set to 1 for males and 0 for females.

The data extracted were reviewed to identify missing data or incomplete data. We removed the papers that did not record at least a proper adoption rate’, even if the determinant variable did exist.

### 2.5 Overview of adoption studies of agricultural technologies

Developing and adopting new agricultural technologies came as a solution for 475 million farmers worldwide, mostly in low- and middle-income countries [37]. This justifies the high number of adoption studies registered in recent decades and the interest of development partners in financing adoption studies. The subject is more pertinent since it mainly involves agricultural decision-makers, especially the end-users and individual households’ beneficiaries of the technologies. Adoption is a determinant of economic growth. The most common indicator is the average adoption rate, estimated as follows:

(1) $$Adoption\;rate\;\left(\%\right)=\frac{x_{i}}{X}*\;100$$

where $x_{i}$ represents the number of farmers who accepted or used technology *i,* and X is the total number of farmers aware of the technology.

Assuming that farmers are rational and aim to maximize an unobserved utility function, adoption is the realized value of an unobserved latent utility estimated through a linear function [38]:

(2) U=yβ+ε

where β is the vector of estimated parameters, ε is the random error term, and y represents the external factors, financial, agricultural management, environmental, behavioral, and socioeconomic elements. The i^it^ farmer adopts if the expected utility of the introduced technology is > 0.

(3) $$x_{i}=\left\{ \begin{array}{c} 1,if\;U_{i}\geq 0\\ 0,otherwise \end{array}\right.$$

where $x_{i}$ is the observed adoption by farmer *i*.

Several other adoption models are used in the literature [38]. However, the studies selected are based on quantitative data collected using structured questionnaires to assess farmer adoption or absence of technology. The notion of adoption is generally perceived as using the technology for a defined period or not. Although all selected studies did not define what they meant by adoption, the mathematical estimation was still the same across studies. In this study, we consider adoption and the rate as described and estimated in the selected studies.

### 2.6 Meta-analysis

The main task is to analyze the effect size and estimate its determining factors. We used the random-effects model based on the assumption that all selected studies are unlikely to be similar, and the goal is to put together the true effect sizes using the weight [39]. Therefore, the weighted average effect size computed by the random effect estimates the weighted mean of a distribution of true effect sizes. The weight of the *i*th effect size is determined as follows:

(4) $$W_{i}=\frac{1}{\left(\tau^{2}+S_{i}^{2}\right)}$$

where $S_{i}^{2}$ represents the within-study variance of study *i*, while $\tau^{2}$ is the between-study variance.

We used Der Simonian–Laird, maximum likelihood (ML), and restricted maximum likelihood (REML) models to estimate the variance $\tau^{2}$ [40].

In addition to the estimation of the effect size based on the adoption rate, we also estimated the partial correlation coefficient (PCC) (and its standard error (SE)) of the socioeconomic characteristics (age, gender, education, farm size, and income) as a measure of the effect size [41]. The PCC and SE were estimated as follows:

(5) $${PCC}_{i}=\frac{t_{i}}{\sqrt{t_{i}^{2}+{df}_{i}}}$$

where $t_{i}$ represents the t value estimated from the variable coefficient and its standard error and ${df}_{i}$ is the degree of freedom of the estimate.

The standard error (SE) of the PCC is estimated as:

(6) $$SE-{PCC}_{i}=\sqrt{\frac{1-{PCC}_{i}^{2}}{{df}_{i}}}$$

Different authors have suggested evaluating the effect size based on the PCC. Cohen [42] suggested small, medium, and large effect sizes with a PCC of 0.1, 0.3, and 0.5, respectively, while Doucouliagos [43] proposed a guideline of 0.07, 0.17, and 0.33 for small, medium, and large effect, respectively. We followed Ogundari and Bolarinwa [44] for a consistent analysis and used both guidelines for this study. We used the absolute value of the PCC to appreciate the effect size of the socioeconomic characteristics identified.

### 2.7 Heterogeneity and meta-regression

Because papers were selected based on a common criterion, variability between the studies chosen is required for the results to be consistent [39,45]. It is expected that studies were different to justify the robustness of the results. It is essential to assess the presence of heterogeneity among the selected studies. This study checked for heterogeneity by using a *Chi*^*2*^ test and P value. These two parameters provided evidence of heterogeneity, and we also used $I^{2}$ to quantify the heterogeneity. I^2^ is the percentage of the total variability due to the true heterogeneity and is estimated as follows:

(7) $$I^{2}=\left(\frac{S-\left(n-1\right)}{S}\right)*100$$

where *S* is the weighted sum of squares of overall studies, and *n* is the number of studies. Based on the percentage (I^2^), the level of heterogeneity was classified as low ($I^{2}$ <25 %), moderate (25 % < $I^{2}$ < 75 %), and high ($I^{2}$ > 75 %) [32]. If the studies revealed low heterogeneity, further analysis was not needed. In the opposite case, we conducted a subgroup analysis to investigate the heterogeneity and minimize the random variations between the point estimate and primary studies. We used the subgroup as described in Section 2.3.

To evaluate the correlation between the overall adoption rate, socioeconomic factors, and publication year, we relied on two methods – fixed effects and random effects, as Stanley and Doucouliagos [43] suggested. The fixed-effects model assumes that all studies have a constant effect size and does not consider that studies differ in terms of sample, model, and specification. In contrast, the random-effects model assumes the effect size distribution across the studies and aims to estimate the mean effect size [46,47]. The true correlation varies across studies and study characteristics [47].

We considered the socioeconomic characteristics of the participants for the studies that make them available. Therefore, we included the Age of the participants, Education, Gender, Income, and Farm size. We included these variables only if they were recorded in the study. In addition, we investigated the correlation between the adoption rate and continents and the trend of the adoption rate to see the interest in publication in relation to the adoption rate of DAT over time. Using the study year would have been interesting, but this information was unavailable for most studies. The significance of the regression coefficient explained how the adoption rate changed with a unit increase in the independent variable. We also regressed the PCC of Age, Gender, Education, Farm size, and Income on the study's characteristics to identify the correlation.

### 2.8 Publication bias

Publication bias is a substantial part of systematic reviews and meta-analyses since it can affect the validity of the study and its generalization [48,49]. That is why it is crucial first to identify the presence of publication bias and then quantify it. The literature used two approaches: the selection model using the weight function to adjust the effect size and the funnel plot approach, which offers a graphical overview, and regression to quantify the bias [48]. In this study, we used the funnel plot approach as it provides a graphical estimation of the bias, offers a formal test of the funnel plot asymmetry with Egger's regression, and fills trim analysis, providing an unbiased effect size estimate. This was the most commonly used approach in several meta-analyses [47,50].

## 3 Results

### 3.1 Characteristics of the included studies and geographic distribution

Table 3 presents the studies that fit the inclusion criteria and their characteristics. The results showed two types of technologies: DAT and precision agriculture. Approximately 75 % of the studies focused on DAT, and 25 % focused on precision agriculture. The data also revealed that the technologies were developed for several reasons, including two fields (91 % crop production and 9 % livestock).

**Table 3:** Includes studies and characteristics.

N'	Authors (Publication year)	Type of Technology	The domain of the Technology	Field	Categories of Access^c^	Use of technology^c^	Subuse Technology^c^	Country
1	Abdullahi et al. (2021)	Precision Tech	Production	Crop production	Access to services	Digital advisory	Smart advisory	Somalia
2	Adrian et al. (2005)	Digital Farming	Extension	Crop production	Access to assets	Smart farming	Equipment monitoring	USA
3	Alam et al. (2018)	Digital Farming	Soil georeferenced sampling	Crop production	Access to services	Digital advisory	Weather information	Bangladesh
4	Ali (2012)	Digital Farming	Information	Crop production	Access to services	Digital advisory	Smart advisory	India
5	Barnes et al. (2019)	Digital Farming	Market information service	Livestock	Access to assets	Smart farming	Equipment monitoring	EU^a^
6	Bolfe et al. (2020)	Digital Farming	Production	Crop production	Access to services	Digital advisory	Smart advisory	Brazil
7	Boyer et al. (2016)	Digital Farming	Web-based	Crop production	Access to assets	Smart farming	Smart shared assets	USA
8	Carillo and Abeni (2020)	Precision Tech	Machine guidance	Crop production	Access to assets	Smart farming	Equipment monitoring	Italy
9	Çetin et al. (2016)	Digital Farming	Crop protection	Crop production	Access to markets	Agri-e-commerce	Inputs and outputs	Turkey
10	Chikuni and Kilima (2019)	Digital Farming	Production	Crop production	Access to markets	Agri-e-commerce	Inputs and outputs	Malawi
11	D'Antoni et al. (2012)	Digital Farming	–	Livestock	Access to assets	Smart farming	Equipment monitoring	USA
12	Daum et al. (2021)	Digital Farming	Input information	Crop production	Access to assets	Smart farming	Smart shared assets	Nigeria
13	Dissanayeke and Wanigasundera (2014)	Digital Farming	Extension	Crop production	Access to services	Digital advisory	Smart advisory	Sri Lankan
14	Drewry et al. (2019)	Digital Farming	Production	Crop production	Access to services	Agri digital financial services	Accountability tool	USA
15	Groher et al. (2020)	Digital Farming	Management	Crop production	Access to assets	Smart farming	Livestock and fishery management	Swiss
16	Hartmann et al. (2020)	Precision Tech	Precision fertilizer, precision tillage, weed management, precision sowing, and sensors	Crop production	–	–	–	Kenya
17	Hay and Pearce (2014)	Digital Farming	Extension	Crop production	Access to assets	Smart farming	Smart shared assets	Australia
18	Hoang (2020)	Digital Farming	Production	Crop production	Access to markets	Agri-e-commerce	Output	Vietnam
19	Kante et al. (2017)	Digital Farming	Extension	Crop production	Access to markets	Agri-e-commerce	Inputs	Mali
20	Kante et al. (2019)	Digital Farming	Information	Crop production	Access to markets	Agri-e-commerce	Inputs and outputs	Mali
21	Karanja et al. (2020)	Precision Tech	Production	Livestock	Access to services	Digital advisory	Smart advisory	Tanzania
22	Kernecker et al. (2020)	Digital Farming	Production	Crop production	Access to assets	Smart farming	Smart shared assets	EU
23	Khan et al. (2019)	Digital Farming	Production	Crop production	Access to services	Digital advisory	Smart advisory	Pakistan
24	Krell et al. (2021)	Precision Tech	Software application	Crop production	Access to markets	Agri-e-commerce	Inputs and outputs	Kenya
25	Larson et al. (2008)	Precision Tech	Mapping, sampling	Crop production	Access to assets	Smart farming	Equipment monitoring	USA
26	Lencsés et al. (2014)	Precision Tech	Variable rate nitrogen technology	Crop production	Access to assets	Smart farming	Equipment monitoring	Hungarian
27	Leng et al. (2020)	Precision Tech	Production	Crop production	Access to services	Digital advisory	Smart advisory	China
28	López-Becerra et al. (2016)	Digital Farming	Production	Crop production	Access to services	Agri digital financial services	–	Spain
29	McCampbell et al. (2021)	Digital Farming	Production	Crop production	Access to services	Digital advisory	Smart advisory	Rwanda
30	Michels et al. (2020)	Digital Farming	Extension	Livestock	Access to services	Digital advisory	Smart advisory	Germany
31	Michels et al. (2020a)	Digital Farming	Input information	Crop production	Access to services	Digital advisory	Pest and disease management	Germany
32	Michels et al. (2020b)	Digital Farming	Production	Crop production	Access to markets	–	–	Germany
33	Mitchell et al. (2018)	Digital Farming	Info crop, financial	Crop production	Access to assets	Smart farming/Precision	Smart shared assets	Canada
34	Mwalupaso et al. (2019)	Digital Farming	Extension	Crop production	Access to services	Digital advisory	Smart advisory	Zambia
35.	Okello et al. (2020)	Digital Farming	Extension	Crop production	Access to markets	Agri-e-commerce	Inputs and outputs	Tanzania
36	Ortiz-Crespo et al. (2020)	Digital Farming	Production	Crop production	Access to services	Digital advisory	Smart advisory	Tanzania
37	Owusu et al. (2017)	Digital Farming	Communication, extension	Crop production	Access to services	Digital advisory	Smart advisory	Ghana
38	Paustian and Theuvsen (2017)	Precision Tech	Autosteer GPS guidance system	Crop production	Access to assets	Smart farming	Equipment monitoring	Germany
39	Pede et al. (2018)	Digital Farming	Management	Crop production	Access to markets	Agri-e-commerce	Inputs	India
40	Pivoto et al. (2019)	Precision Tech	Remote sensing	Crop production	Access to assets	Smart farming	Smart shared assets	Brazil
41	Raheem (2020)	Digital Farming	Digital finance	Crop production	Access to services	Digital advisory	Smart advisory	Australia
42	Rajkhowa Id and Qaim Id (2021)	Digital Farming	Decision support	Crop production	Access to services	Digital advisory	Smart advisory	India
43	Schulz et al. (2021)	Digital Farming	Extension	Crop production	Access to services	Digital advisory	Smart advisory	Australia
44	Sheng and Lu (2020)	Digital Farming	Agribusiness	Crop production	Access to markets	Agri-e-commerce	Inputs and outputs	China
45	Tamirat et al. (2017)	Digital Farming	Marketing	Crop production	Access to assets	Smart farming	Smart shared assets	EU^b^
46	Thar et al. (2021)	Digital Farming	Market information service	Crop production	Access to services	Digital advisory	Smart advisory	Myanmar
47	Vecchio et al. (2020)	Precision Tech	Precision soil sample tool	Crop production	Access to services	Digital advisory	Product verification	Italia
48	Voss et al. (2021)	Digital Farming	Production	Crop production	Access to services	Digital advisory	Smart advisory	Senegal
59	Walton et al. (2008)	Digital Farming	Market information service	Crop production	Access to markets	Agri-e-commerce	Inputs and outputs	USA
50	Yoon et al. (2020)	Digital Farming	Smart farm	Crop production	Access to services	Digital advisory	Smart advisory	Korea
51	Yu et al. (2020)	Digital Farming	Extension	Crop production	Access to markets	Agri-e-commerce	Inputs and outputs	China
52	Zheng and Ma (2021)	Digital Farming	Agri info Extension	Crop production	Access to markets	Agri-e-commerce	Inputs and outputs	China

a Belgium, Germany, Greece, the Netherlands.

b Denmark/Germany.

c Classification by Abbie [35].

**Fig. 2:**
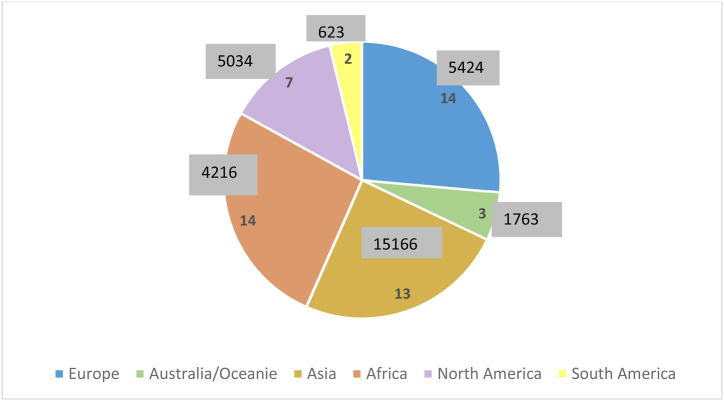
Distribution of the studies by continent.

**Fig. 3:**
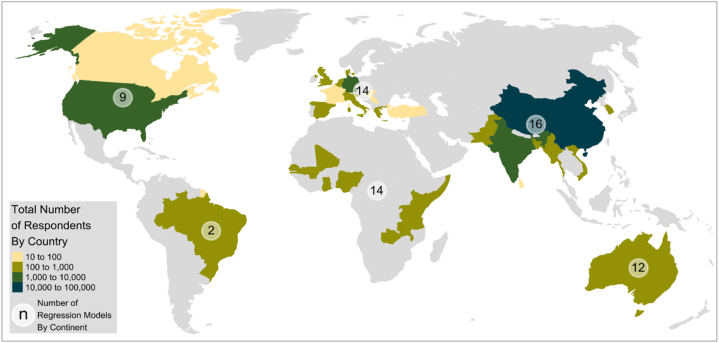
Geographic distribution of the number of regression models.

Fig. 2 shows that the study covered six continents, including Africa (14 studies), Europe (14 studies), and Asia (13 studies). However, Asia had the highest number of events/participants (15,166), representing 89 % of the total. Fig. 3 presents an overview of the distribution of the selected studies and the number of regression models on the map. It showed how DAT spread around the world. Technological diversity and worldwide spread explain DAT’ importance and usefulness.

### 3.2 Meta-analysis results

#### 3.2.1 Overall pooled adoption rate and subgroup analysis

**Fig. 4:**
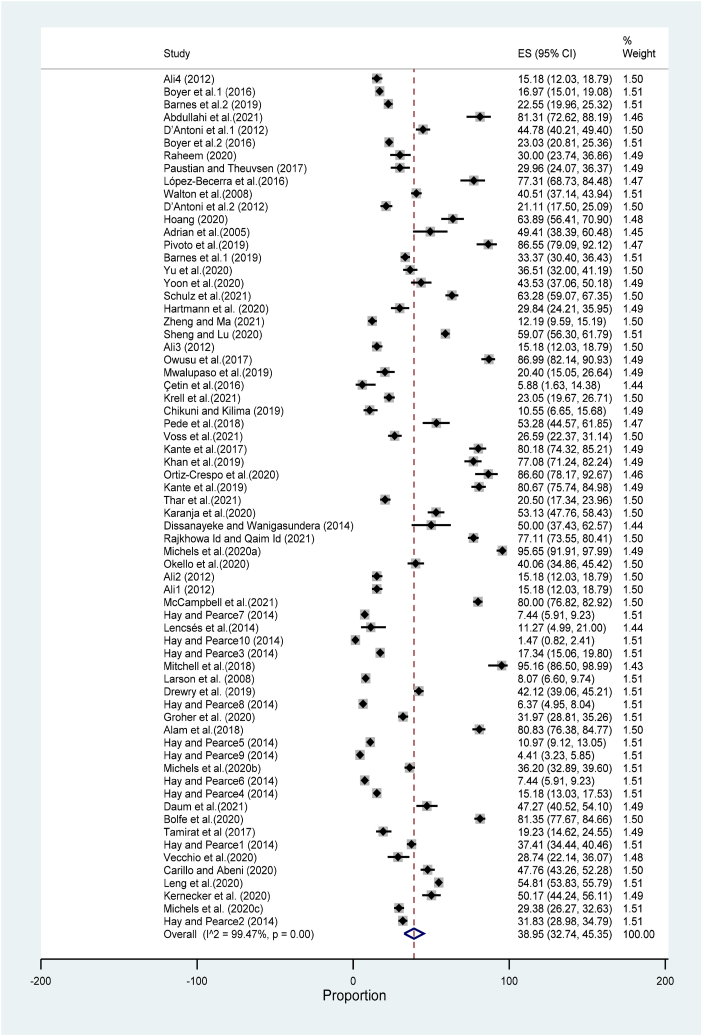
Overall pooled effect size (ES) summary.

Fig. 4 summarizes the pooled adoption rate of 52 (Supplemental S2) overall studies with 32400 participants worldwide. The results revealed that the pooled digital farming technology adoption rate was 38.95 % (CI: 32.74, 45.35). High heterogeneity across the studies was observed (I^2^ = 99.47 %, P value ≤ 0.001). This confirms the variability among socioeconomic characteristics, countries, and technologies. We investigated the heterogeneity by conducting a subgroup analysis. The results showed that when comparing the continents, South America had the highest adoption rate, 82.45 % (CI: 79.34, 85.36), with 623 participants, followed by Africa, 53.73 % (CI: 38.12, 68.98) with 4216 participants (Supplemental Figure S3). The adoption rate of the technologies developed for the extension was the highest at 46.79 % (CI: 34.87, 58.89). Although there was a similar adoption rate for technologies developed related to crop production (ES: 39.11, CI: 32.44, 45.99) and livestock (ES: 37.43, CI: 20.59, 56.00), there was a high number of published papers in agriculture compared to livestock. Regarding the type of technology, digital farming technology had the highest adoption rate: 48.42 % (CI: 41.71, 55.18).

Moreover, the results of the subgroup analysis based on the classification by Abbie [35] showed that in the categories of access, service access technologies had the largest adoption rate: 52.20 % (CI: 42.76, 61.56), followed by access to market technologies with an adoption rate of 40.55 % (CI: 28.72, 52.95) (Supplemental Figure S3). Regarding the category of the use of the technologies, the results revealed that digital advisory technologies had the highest adoption rate: 51.55 % (CI: 41.33, 61.70), followed by agri-e-commerce technologies with an adoption rate of 40.90 % (CI: 27.66, 54.85).

#### 3.2.2 Meta-regression

The multivariate meta-regression by continent revealed that all six continents were significant. This result shows an overall tendency to adopt digital farming technologies worldwide. The highest mean adoption rate was in South America, at 83.8 %. However, it had the smallest number of participants (623) from only two studies, and the adoption rate record could be due to the impact of small sample studies. This justifies why we investigate the effect of the small sample studies on the adoption rate. Africa was the second continent, with a mean adoption rate of 53 % (Table 4).

**Table 4:** Meta-regression of the continents.

	Coefficient	Standard Error
Africa	0.530***	0.064
Asia	0.428***	0.059
Australia	0.193***	0.067
Europe	0.373***	0.064
North America	0.362***	0.079
South America	0.838***	0.171

I^2^ (%) = 96.85.

Prob > chi2 = 0.000.

R-squared (%) = 2.

N = 67.

Test of residual homogeneity: Q_res = chi2(62) = 1353.63 Prob > Q_res = 0.000.

***1 % significant; **5 % significant; and *10 % significant.

**Fig. 5:**
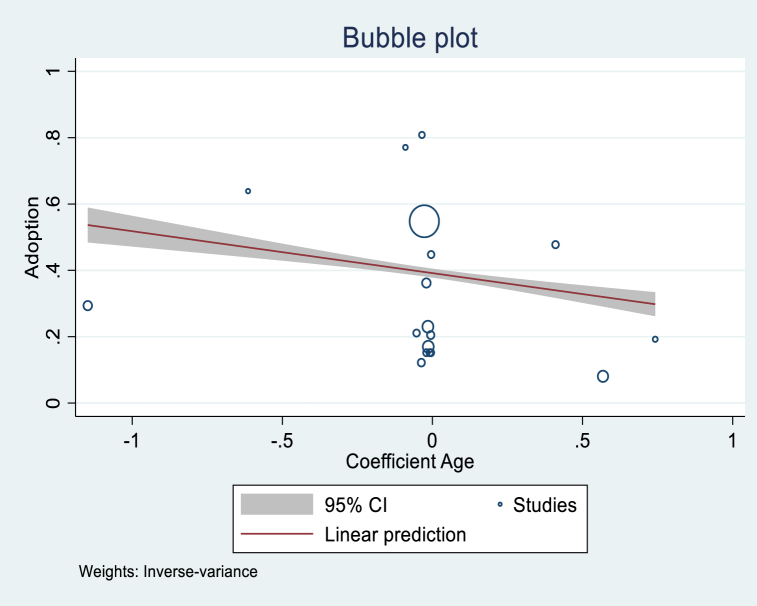
Shape of correlation Age coefficient-Adoption.

**Fig. 6:**
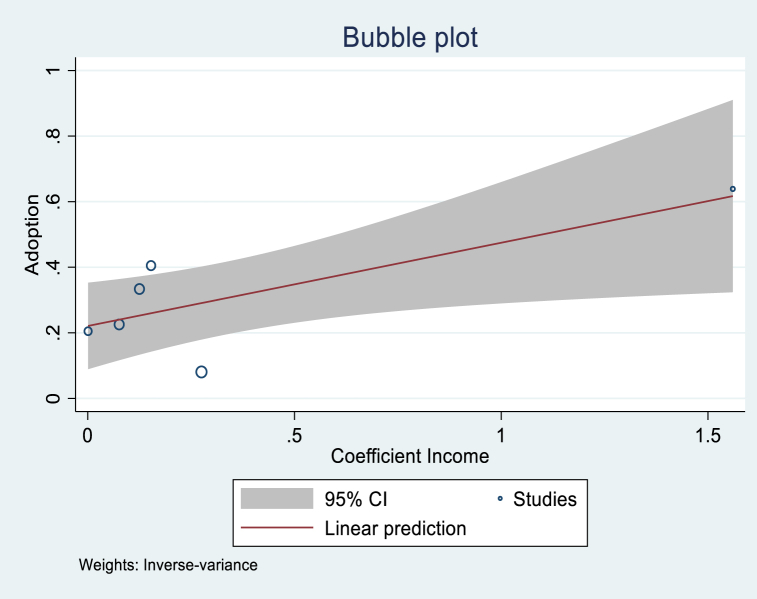
Shape of correlation Gender coefficient-Adoption.

**Fig. 7:**
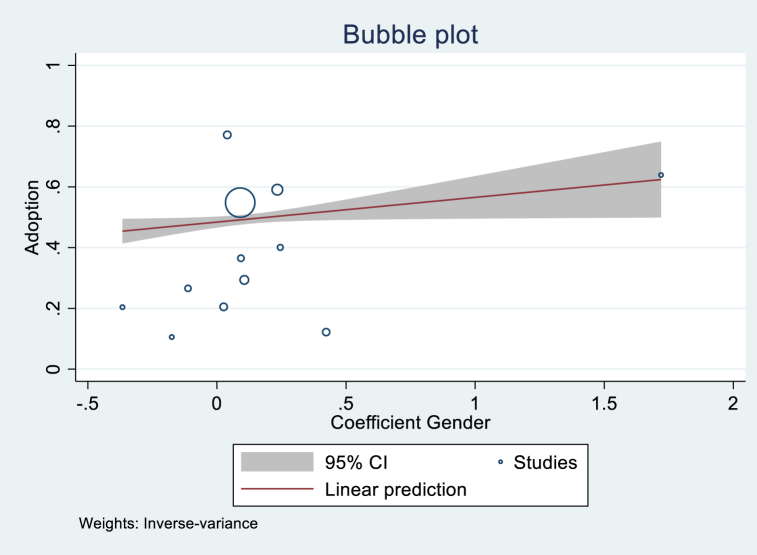
Shape of correlation Income coefficient-Adoption.

**Fig. 8:**
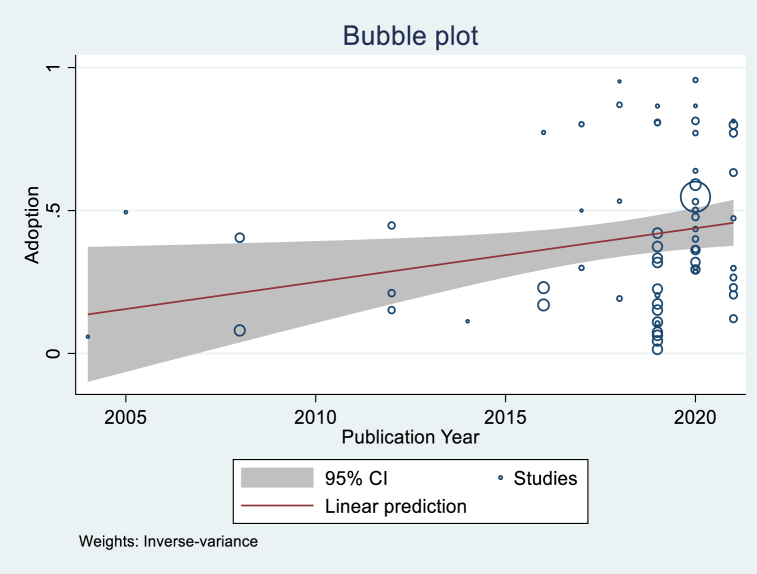
Shape of correlation Publication year -Adoption.

Table 5 shows the regression analytical results, showing that the variables of Age, Gender, Income, and Publication year were significant. The variable Age had a coefficient of −0.054 and was significant at 1 %. This implies that studies that found more positive age effects tended to have lower overall adoption rates. This result could be explained by the interest of young farmers and their accessibility to new technologies. Even if we assume that older farmers have more experience, digital technologies require a minimum knowledge of ICT tools, and young farmers have more chances to acquire those skills. Even though not significant, the variable Farm size was positive, implying that the studies with positive farm size tended to have greater overall adoption rates. This is an expected sign since we can assume that the larger the farm, the more important it is to improve the management of the resources to reduce losses along the production chain and maximize profit. In general, male farmers tend to adopt DAT more than female farmers, according to the significant and positive variable Gender in the adoption of digital technologies. As expected, the variable Income was positive and significant, implying that the purchasing power of farmers plays a determinant role in the adoption of digital farming technologies. Publication year was also positive and significant. Fig. 5, Fig. 6, Fig. 7, Fig. 8 illustrate the shape of the adoption rate for these four variables.

**Table 5:** Random effect meta-regression analysis.

	Coefficient (Standard Error)	Constant	Prob > chi2
Age (N = 25)	−0.054***(0.016)	0.400	0.000
Farm size (N = 16)	0.028 (0.063)	0.313	0.657
Education level (N = 19)	−0.543 (0.389)	0.453	0.163
Gender (N = 12)	0.081**(0.039)	0.484	0.039
Income (N = 6)	0.253**(0.112)	0.220	0.023
Publication year (N = 67)	0.018**(0.008)	−37.607	0.024

***1 % significant; **5 % significant; and *10 % significant.

#### 3.2.3 Effect of small sample studies on the overall adoption rate

**Fig. 9:**
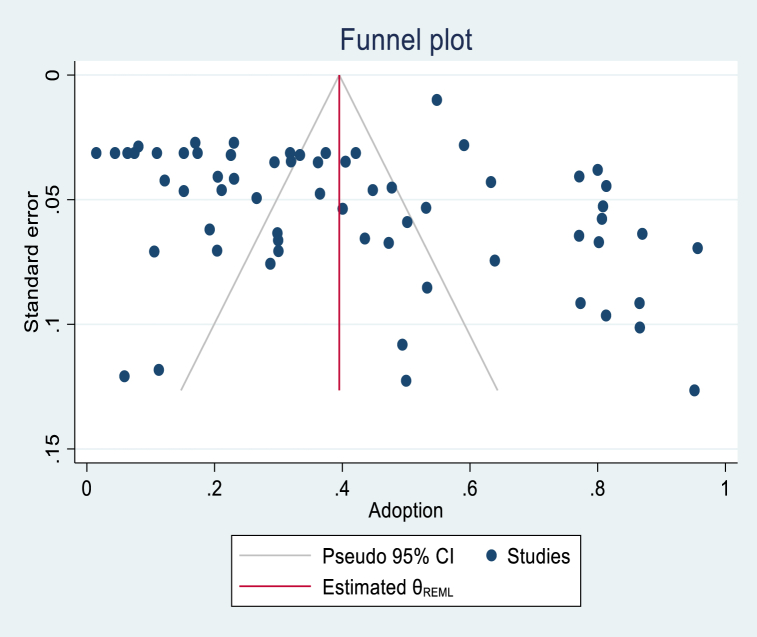
Overall funnel plot.

**Fig. 10:**
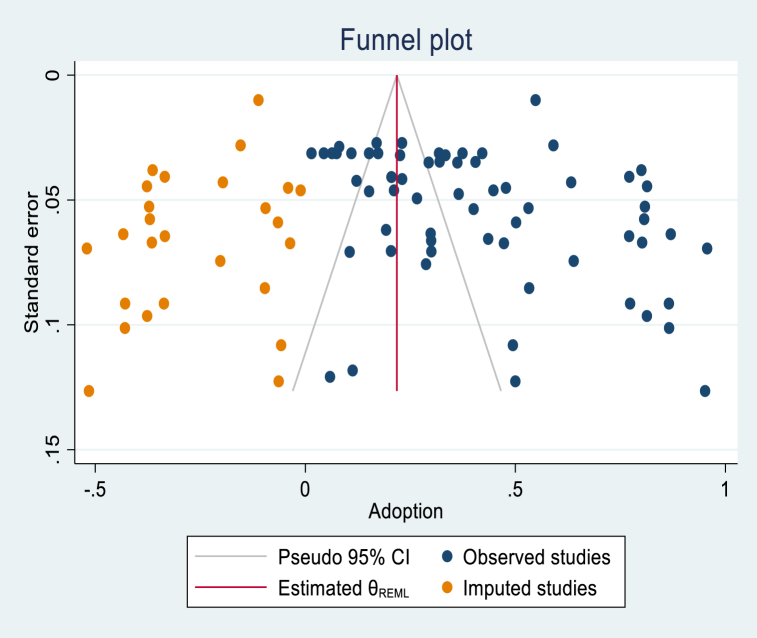
Trim and fill funnel plot.

The graphical assessment of the funnel plot suggested a likely presence of publication bias due to the small sample studies (Fig. 9). The red horizontal line indicates the weighted average effect size across studies. The asymmetry funnel plot confirms the presence of publication bias, which could also be due to the high heterogeneity among studies and small sample studies. To investigate this hypothesis, the funnel plot by group (Continents, Type of technology, Field, and Technology use) showed an asymmetric funnel plot for the different groups (Supplemental Figure S4). This confirmed that the funnel plot asymmetry is likely not due to the heterogeneity between studies but to the publication bias of small sample studies. The Egger's test confirmed this result and revealed a statistically significant publication bias (p < 0.001) (Supplemental S5). The trim and fill analysis (Fig. 10) showed 26 studies (pulled on the left side of the graph; this does not mean that the adoption of those missing studies was negative), potentially missing studies from the meta-analysis due to publication bias. Using the observed and imputed studies, the computed overall adoption rate was 21.9 % (CI: 14.1, 29.5) if the missing studies were included.

#### 3.2.4 PCC of socioeconomic factors and correlation with technology characteristics

The results of the meta-analysis (Supplemental Figure S6) show that when the studies are taken individually for the variable Age, only one study out of twenty-two had a large effect size (PCC greater than 0.5) according to the Cohen guideline, and the remaining are 0.00 <PCC<0.3. However, considering the Doucouliagos guideline, seven studies out of 22 had a PCC lower than 0.07, and only one had a PCC greater than 0.33. For the variable Gender, two out of 11 studies had a PCC greater than 0.1; therefore, Cohen considered it a small effect size. We counted four studies with PCCs greater than 0.07, considered a small effect size, and two studies with PCCs greater than 0.17, according to Doucouliagos. Regarding the variable Education, we counted six studies with a small effect size and only one study with a medium effect following Cohen. When considering the Doucouliagos guideline, eight studies had a small effect size, and two had a medium effect size. For the farm size variable, four out of twelve studies had a small effect size, one study had a medium effect, and one had a large effect when using the Cohen guideline. These results show only one study with a small effect size, according to Cohen, and a medium effect, according to Doucouliagos.

**Fig. 11:**
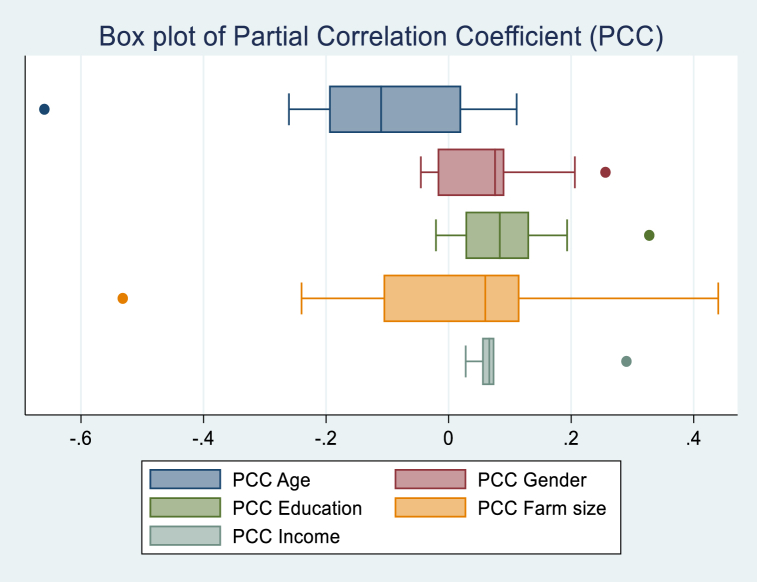
Box plot PCCs of socioeconomic variables.

The estimation of the average PCC (Table 6) shows that Age and Income had the largest PCC with 0.103, while farm size had the lowest with 0.001. The distribution of PCC results in Fig. 11 shows a large amount of variability in the PCC of farm size. When stratifying the PCCs, Table 5 shows that according to Cohen's guideline, Age and Income had a small effect size. At the same time, this variable was classified as a medium effect when referring to the Doucouliagos guideline.

**Table 6:** PCC stratification by Cohen and Doucouliagos.

PCC	Observation	Mean	Standard Deviation	Cohen	Doucouliagos
Age	22	−0.103	0.168	Small	Small
Gender	11	0.072	0.093	–	Small
Education	17	0.094	0.087	–	Small
Farm Size	12	0.001	0.239	–	–
Income	5	0.103	0.106	Small	Small

The high values of the I^2^ statistic identified in all variable meta-analyses showed variability in the results due to study differences. We explored heterogeneity using meta-regression with the study characteristics. Table 7 shows no significant correlation between the PCC of the socioeconomic variables and the publication year.

**Table 7:** Meta-regression of PCCs and study characteristics.

	Age (22^a^)	Gender (11^a^)	Education (17^a^)	Farm size (12^a^)	Income (5^a^)
Coefficient (Standard Error)					
Publication Year	−0.003 (0.008)	0.032 (0.043)	0.003 (0.005)	−0.010 (0.018)	0.009 (0.008)
Constant	7.234 (16.696)	−64.573 (86.623)	−4.976 (0.005)	20.151 (36.400)	−19.906 (16.139)
Prob > chi2	0.6603	0.4555	0.595	0.5799	0.2151
R-squared (%)	0.00	0.00	0.00	0.00	11.80
Technology Domain					
Extension	−0.085 (0.055)	0.057 (0.036)	0.073**(0.030)	0.167 (0.138)	–
Management	−0.098 (0.078)	0.144**(0.068)	0.066 (0.042)	−0.017 (0.138)	0.121**(0.0563)
Production	−0.130**(0.066)	0.053 (0.068)	0.150***(0.038)	−0.073 (0.097)	0.028 (0.112)
Prob > chi2	0.0499	0.0550	0.000	0.5597	0.0952
R-squared (%)	0.00	0.00	7.03	0.56	0.00
Type of technology					
Digital Farming	−0.142***(0.039)	0.071**(0.027)	0.108***(0.024)	0.025 (0.094)	0.290***(0.021)
Precision Technology	0.003 (0.064)	–	0.050 (0.043)	−0.032 (0.111)	0.055***(0.010)
Prob > chi2	0.0014	0.0102	0.000	0.9261	0.000
R-squared (%)	11.64	–	2.16	0.00	96.33
Field					
Crop production	−0.116***(0.037)	0.073**(0.031)	0.101***(0.021)	0.001 (0.069)	0.102**(0.047)
Livestock	0.033 (0.118)	0.058 (0.098)	−0.021 (0.085)	–	–
Prob > chi2	0.0072	0.050	0.000	0.9911	0.030
R-squared (%)	2.19	0.00	5.61	–	–
Categories of Access					
Access to assets	0.001 (0.070)	–	0.036 (0.049)	−0.032 (0.117)	0.056 (0.057)
Access to markets	−0.063 (0.059)	0.111***(0.035)	0.079**(0.035)	0.044 (0.151)	0.171**(0.069)
Access to services	−0.182***(0.050)	0.025 (0.038)	0.127***(0.030)	0.010 (0.131)	–
Prob > chi2	0.0021	0.005	0.000	0.9827	0.0282
R-squared (%)	13.22	14.68	4.51	0.00	14.37
Use of the technology					
Agri-e-commerce	−0.052 (0.065)	0.111***(0.035)	0.078**(0.040)	0.031 (0.196)	0.173**(0.07)
Digital advisory	−0.182***(0.050)	0.025 (0.038)	0.127***(0.031)	0.010 (0.139)	–
Smart farming	0.001 (0.072)	–	0.036 (0.051)	−0.032 (0.124)	0.056 (0.057)
Prob > chi2	0.0065	0.005	0.000	0.9969	0.028
R-squared (%)	9.37	14.68	0.00	0.00	14.37

***1 % significant; **5 % significant; and *10 % significant.

a We have fewer observations here because not all studies provided the standard error. We then estimated the PCCs for the studies with coefficients and standard errors.

The meta-regression found a negative and significant correlation between the effect size of Age and the technologies related to production, digital farming, crop production, access to service, and digital advisory. This correlation indicates that younger farmers are more likely to adopt the technologies.

We also found that the effect size of gender was positively correlated with adopting DAT related to management, digital farming, crop production, access to the market, and e-commerce. These results indicate that the role of male farmers is an important factor that needs to be considered in the dissemination and adoption process.

A positive correlation was found between the PCC of Education and the technologies related to extension, production, digital farming, crop production, market access, services, e-commerce, and digital advisory. This implies that farmers with higher education levels are more susceptible to adopting digital farming technologies, especially technologies related to extension, production, digital farming, and crop production e-tools, which facilitate access to the market and services and provide digital personalization advisories.

As expected, the results also showed a positive correlation between the effect size of Income and the adoption of digital farming technologies related to production, precision technology, crop production, access to the market, and e-commerce. In any case, the use of digital technology would be free of charge, even if the farmer is a direct user; they will need to have adequate infrastructure and require a minimum investment. However, higher-income farmers are simply more likely to be tech-savvy and, thus, more likely to adopt DAT.

## 4 Discussion

Numerous research and development initiatives have focused on emerging digital technologies and their critical role in agricultural development and economic progress [[51], [52], [53]]. However, several questions still need to be answered to achieve the expected impact. This study followed the PRISMA protocol and proposed a systematic review to obtain an overall view of adopting DAT. The review showed an average adoption rate of 38.95 %. However, heterogeneity was found across the studies. The variabilities could be explained by the difference in the socioeconomic characteristics, economic status of the country or continent, the need in terms of technology, or the technology itself. Overall, 38.95 % is an accepted adoption rate for the following reasons. First, even though digital technologies are designed for everyone, especially open-source technologies, not all users, particularly farmers in rural areas, can access them. This could negatively affect the overall adoption rate of these technologies. Second, some technologies are well designed but hard to use or useless for farmers, hence leading to low adoption and/or quick dis-adoption. Subgroup analysis showed that South America and Africa, which included most low-income countries, have the highest adoption rates. However, given the few studies recorded in South America (2 studies), we assume that this is not enough to generalize. Sabi et al. [54] used the Technology Acceptance Model (TAM) and found that socioeconomic background is the main factor driving technology adoption in Africa. Other studies estimated the adoption of DAT in African countries [3,[55], [56], [57]]. The interest could be explained by the ability of farmers in developing countries to overtake traditional practices in favor of new technology adoption [12] or by the several agricultural projects and new policies being implemented in these continents for conversion toward modern agriculture. This result confirms the study conducted by Nowak [58] in developed countries, which shows a higher adoption rate of 60–80 %. However, the use of digital agricultural technology was still relatively low in developing countries compared to high-income countries [59]. This is confirmed by Trendov et al. [12].

Regarding the type of DAT, the review showed that extension technology is the most important. This result should attract the attention of policymakers and investors in the private sector who are interested in digital advisory. For example, in partnership with Precision Agriculture Development (PAD), in 2021, the International Fund for Agricultural Development (10.13039/100008687IFAD) launched a project to provide personalized agricultural advice to 1.7 million small-scale farmers through mobile phones in Kenya, Nigeria, and Pakistan [60]. Several digital advisory services and applications were developed to provide farmers with quality and more efficient information [61,62]. The digital extension service covered both crop production and livestock. However, there is more interest in crop production, demonstrated by the low number of published papers on livestock. Our findings aligned with Shang et al. [29], who removed livestock papers from the systematic review due to the limited number of articles available.

The acceptance and adoption of DAT could be seen as an opportunity for the younger generation to invest in agriculture. Our findings go in this direction and could be explained by several factors favorable to young people, such as accessibility to new technologies, the use of new technologies, and accessibility to information. Even with a low e-literacy, young farmers were more open to innovation and were more likely to be familiar with digital technology [63]. This finding is in line with that of Czaja et al. [64] and Penard et al. [65], who also found that adults were less likely to adopt new technologies than young adults who are susceptible to being educated and, therefore, more open to ICT use and adoption. However, older people would experience a better quality of life, income, and wellbeing if they used the new technologies [66], including DAT with the appropriate business model [21,67,68]. This is open for discussion since e-literacy, access to information, and knowledge of ICT tools differ from one continent and country to another. According to a 2016 United Nations Education, Scientific, and Cultural Organization report, e-literacy is important for the knowledge economy and information society [69]. The European Commission argued that e-literacy had become an essential life competence, and its inability could become a barrier to social integration and personal development [70]. High-income countries have better access to ICT and better e-literacy. Therefore, this is not a barrier to adopting DAT. Our review also revealed that the size of the farm is a determinant factor and has a positive effect on the decision to adopt digital agricultural technology. This means wealthy farmers have better access to digital tools and better e-literacy. Another issue to consider is that some technologies, especially the precision agricultural technologies in the Global North, require an initial investment or payment of a recurrent from farmers. As a farm grows, it becomes more crucial to utilize its resources effectively to minimize losses and expenses and increase profit. Digital technologies and precision tools could be the appropriate solution to achieve this. This means that larger farms will find it easier to adapt, but small-scale farmers may lag. Blasch et al. [71] also found that farm size and economies of scale are crucial for adoption since larger farms value DAT more than small farms. The authors found that small farms value less fertilizer saving, water quality improvement, and personalized advice than farmers of large farms. This explains why precision tools are more adopted in high-income countries since they have an intensive agricultural production system and value DAT. This result aligns with Shang et al. [29], who found that farm size and education positively affect farmers’ decisions to adopt DAT. When assessed from an opposite view, if we consider that small farms are under more pressure as they have fewer or limited resources and manage to use them more efficiently. Smaller farms are exposed to more risk aversion, as any slight change in farming practice may imperil their food security; therefore, they are less likely to be early adopters.

With the impact of adopting different DAT and considering the barriers, the adoption rate has increased over the years. This may result from implementing many projects, service-based business development, infrastructure development (more farmers live in areas with network coverage, more farmers have access to electricity, or more farmers own phones), and farmers' willingness to adopt. However, the adoption rate is still low, notwithstanding the efforts, which is why further strategies, policies, and business models are needed [68] (lack of ICT skills, financial support, lack of infrastructure, etc.) to overcome the barriers to adoption [72]. The meta-regression also showed that although all six continents were significant, there was a difference in the adoption rate tendency. South America has the highest mean adoption rate, implying a positive adoption tendency of DAT. Note that we registered only two papers in South America, which may be too small to generalize. However, this result provided an overview of farmers’ ability to adopt DAT. The finding also reveals that low- and middle-income continents (Africa, Asia, and South America) have an adoption rate higher than high-income continents (Australia, North America, and Europe) and are currently the most open to adopting the technologies. This could be explained by the fact that the adoption of DAT is not a great challenge for high-income continents as it is in low and middle continents, as seen through the number of papers registered in these continents, which is relatively lower compared to low and middle continents. 10.13039/100014337Furthermore, this could be explained by the number of ongoing projects and technology developments supporting DAT and their dissemination in Africa. Both research and development partners are working through technology development, start-up funding, technical support, and policymakers for technology dissemination. It is worth noting that the high-income continents are where we registered the lowest adoption rate tendency compared to the low and middle-income continents. This information is relevant, especially for private investors searching for agribusiness opportunities or technology developers searching for an appropriate environment to introduce new technology. In addition, the overall 39 % adoption rate is a ballpark figure that can help investors in private digital farming initiatives estimate the return on investment. It is also crucial for policymakers to develop policies and strategies to support the development and introduction of new technologies favoring farmers.

The results of the socioeconomic variable PCCs meta-analysis allow us to appreciate how large or small the effect size is. Gender plays a determinant role in the adoption process. Even though the study shows that male farmers have more access and are more likely to adopt DAT, the role of females is still essential, and they should also be involved in the adoption process. Female involvement is suggested to be from technology development to dissemination using their voices and channels to reduce the gender gap in adopting DAT [56] or by applying systematic gender-inclusive participatory design methodologies [73]. These findings align with those of Kinkingninhoun Medagbe et al. [74], who also established gender inequality where men have more access to technology and are more likely to adopt technology information and knowledge in West Africa. The effect sizes of the level of education and income were also positively correlated with adopting DAT. Education is seen as an essential factor in facilitating e-literacy and the adoption of DAT. Suggesting that many DAT and services are not yet fully inclusive to farmers with low levels of education. More could be done to support adoption by these farmers, for example, by avoiding the need for literacy or including a literacy program in the dissemination plan. In some cases, extension services appear to substitute for formal education in promoting adoption [38], indicating that farmers with less education need more support and training to learn the importance of using DAT. On the other hand, farmers willingness to use technology and their level of education or e-literacy and purchasing power are critical factors to adaptation since the direct use of DAT requires a minimum infrastructure (devices, network, electricity, etc.), which needs to be acquired by the end user.

## 5 Conclusion and implications

Global development, particularly agriculture, requires modern solutions to meet economic development expectations' challenges and ensure long-term development. This research contributes by shedding light on the key development tools that have increased in the last decade. It provides a global perspective on adopting DAT, including evidence of each continent's global adoption rate and determinants. It also includes information on adopters' behavior and the factors influencing the adoption of various DAT. We believe that developing and disseminating DAT must be accompanied by a business model that considers end-users socioeconomic characteristics and potential.

The question of adoption, which comes after technology development and dissemination, is one that research, development, and decision-makers are paying close attention to. There is a need to answer the question: How is the global uptake of digital agriculture technology going? What variations in adoption are there when socioeconomic characteristics are considered? What factors can technology developers base their designs on to make them more suitable in the future? and What effects do socioeconomic factors have on the uptake of various technologies? By gathering quantitative information on the rate of adoption and determinant factors of technologies adopted by farmers, the study attempted to answer these questions.

DAT could play a significant role in agricultural and sustainable development and contribute to farmers' wealth. However, adoption is still controversial, especially in developing countries with low purchasing power and low e-literacy. Nevertheless, this does not make it impossible to find an appropriate solution that fits each country's socioeconomic reality. This study proposed a worldwide overview of the adoption of agricultural digital technologies and the determinants for the first time. Based on the studies identified, the data collected, and the analysis, the study derived the following main points.

- The study found a clear interest in adopting DAT and its pertinence defined by farmers' willingness to use the technologies through the papers reviewed. We found 39 % and 22 % adoption rates when considering the potentially missing studies. Africa and South America were the two continents that proved to have the highest adoption rates.
- We also found a negative correlation between the adoption rate and Age, which indicates that younger farmers adopt more. A positive correlation between publication year and the adoption rate shows the interest and increase in adopting agricultural digital technologies research.
- The studies also revealed a positive correlation between gender and income, implying their importance in adoption.
- A significant correlation was found between the effect size of the socioeconomic variables (age, gender, education, and income) and the adoption of DAT related to production, management, digital farming, crop production, access to markets and services, and digital advisory.

The study's findings are relevant and valuable to policymakers, private investors, and the academic community. Using the findings on the most adopted type of technology, policymakers, and private investors could make better decisions on the type of technology to develop and promote. This information is also crucial for the academic community as it provides new and quantitative findings to the existing literature on adopting DAT. Furthermore, the characteristics that drive the adoption are also important factors that policymakers could use to design a better and sustainable adoption approach. Private investors can also use it to design tailor-made technology with a higher probability of adoption. Nevertheless, the role of policymakers and external partners is crucial in supporting innovation and encouraging the use of DAT by end-users and extension agents.

## 6 Limitations and future studies

Within this study, we adhered to the PRISMA protocol to provide accuracy and a more realistic result. This paper has some limitations. First, because of the outcomes reported and the nature of the selected studies, we did not assess the certainty of evidence. Second, only the technology created for agricultural purposes was considered digital agricultural technology. Therefore, this excludes digital technologies or devices not intended for agricultural use but are nonetheless used for that purpose, such as cellphones, smartphones, tablets, software, computers used for agriculture, precision tools, television, and radio. Therefore, a study that includes all those layers could have different results. Third, the papers included in the study were found using only Scopus and Web of Science and were based on pre-defined keywords. Different keyword searches and other search tools, like "Google Scholar," may yield different input studies, leading to different results. Therefore, any research paper on the adoption and determinants not included in these databases may have gone unnoticed. However, the challenge here was to find papers that assess both the adoption and the determinants, which was not the case for most papers initially found. This may be a reason as to why the literature reviewing the adoption is mostly qualitative and does not include the determinants. Lastly, some residual heterogeneity could not be quantitatively explained despite our use of meta-regression, subgroup analysis, and partial coefficient correlation to examine the heterogeneity of the included studies. This heterogeneity was most likely caused by variations in the adoption rates of different technologies between the various countries and when surveys were conducted. However, this does not affect the accuracy of our findings because we explored heterogeneity using suitable methodologies.

Future research may examine digital technologies that aren't intended for agriculture but are nonetheless utilized for agricultural purposes. Additionally, another research search engine, like Google Scholar, might be added to the search database to increase the probability of finding appropriate published papers.

## Funding statement

We thank all 10.13039/501100015815CGIAR Trust Fund and the Government of Belgium for their financial contribution through the Transforming Agrifood Systems in West and Central Africa Initiative (TAFS-WCA). We also thank The International Fund for Agricultural Development (10.13039/100008687IFAD) for financing the project “Sustainable and Diversified Rice-based Farming Systems” (DCI-10.13039/100010678FOOD/2015/360–968). The authors would also like to thank the Excellency in Agronomy (EiA) initiative and the Internal Grant Agency of the Faculty of Tropical AgriSciences: [Grant Number 20233101].

## Data availability

Data will be made available on request.

## CRediT authorship contribution statement

**Rico Amoussouhoui:** Writing – original draft, Methodology, Investigation, Formal analysis, Data curation. **Aminou Arouna:** Validation, Supervision, Methodology, Conceptualization. **Sacha Ruzzante:** Validation, Software, Methodology, Data curation. **Jan Banout:** Writing – review & editing, Supervision, Funding acquisition, Conceptualization.

## Declaration of competing interest

The authors declare that they have no known competing financial interests or personal relationships that could have appeared to influence the work reported in this paper.
