# Supplementary material for: Adoption of ICT4D and its determinants: A systematic review and meta-analysis
Source: Heliyon. 2024 Apr 25;10(9):e30210. doi: 10.1016/j.heliyon.2024.e30210 (PMC11061747; doi:10.1016/j.heliyon.2024.e30210)
Supplement: Multimedia component 1 [file mmc1.zip › Supplementary_Files04-12.rtf]

Adoption of ICT4D and its determinants: A systematic review and meta-analysis

Supplemental Materials

Supplemental S1: Search strategies
Search Strategy 1: Web of Science and SCOPUS
''Digital farming technologies''; “Adoption digital agricultural technology”; “Adoption digital farming technology”; ''Digital agricultural technologies''; ''Agriculture digital technology adoption''; ''ICT adoption in agriculture''; ''Determinant adoption ICT agriculture''; ''Determinant adoption digital technologies agricultural'', ''Determinant adoption digital farming technologies''. 
Search Strategy 2: Web of Science and SCOPUS
''Mobile App adoption in agriculture''; ''Adoption digital extension service in agriculture''; ''Factors determining adoption of digital farming technologies''.


Supplemental S2: Reference of the included papers
Abdullahi, Husein Osman, Abdikarim Abi Hassan, Murni Mahmud, and Abdifatah Farah Ali. 2021. Determinants of ICT adoption among small scale agribusiness enterprises in somalia. International Journal of Engineering Trends and Technology - IJETT 69. Seventh Sense Research Group SSRG: 68–76. https://doi.org/10.14445/22315381/IJETT-V69I2P210.
Adrian, Anne Mims, Shannon H. Norwood, and Paul L. Mask. 2005. Producers' perceptions and attitudes toward precision agriculture technologies. Computers and Electronics in Agriculture 48. Elsevier: 256–271. https://doi.org/10.1016/J.COMPAG.2005.04.004.
Alam, GM Monirul, Khorshed Alam, Shahbaz Mushtaq, Most Nilufa Khatun, and MS Arifeen Khan Mamun. 2018. Influence of socio-demographic factors on mobile phone adoption in rural Bangladesh: Policy implications: Information Development 35. SAGE PublicationsSage UK: London, England: 739–748. https://doi.org/10.1177/0266666918792040.
Ali, Jabir. 2012. Factors Affecting the Adoption of Information and Communication Technologies (ICTs) for Farming Decisions. Journal of Agriculture and Food Information 13.  Taylor & Francis Group : 78–96. https://doi.org/10.1080/10496505.2012.636980.
Barnes, A. P., I. Soto, V. Eory, B. Beck, A. Balafoutis, B. Sánchez, J. Vangeyte, S. Fountas, T. van der Wal, and M. Gómez-Barbero. 2019. Exploring the adoption of precision agricultural technologies: A cross regional study of EU farmers. Land Use Policy 80. Pergamon: 163–174. https://doi.org/10.1016/J.LANDUSEPOL.2018.10.004.
Bolfe, Édson Luis, Lúcio André de Castro Jorge, Ieda Del'Arco Sanches, Ariovaldo Luchiari Júnior, Cinthia Cabral da Costa, Daniel de Castro Victoria, Ricardo Yassushi Inamasu, Célia Regina Grego, Victor Rodrigues Ferreira, and Andrea Restrepo Ramirez. 2020. Precision and Digital Agriculture: Adoption of Technologies and Perception of Brazilian Farmers. Agriculture 10. Multidisciplinary Digital Publishing Institute. https://doi.org/10.3390/AGRICULTURE10120653.
Boyer, Christopher N., Dayton M. Lambert, Margarita Velandia, Burton C. English, Roland K. Roberts, James A. Larson, Sherry L. Larkin, Krishna P. Paudel, and Jeanne M. Reeves. 2016. Cotton Producer Awareness and Participation in Cost-Sharing Programs for Precision Nutrient-Management Technology. Journal of Agricultural and Resource Economics 41. Colorado State University: 81–96. https://doi.org/10.22004/AG.ECON.230774.
Carillo, Felicetta, and Fabio Abeni. 2020. An Estimate of the Effects from Precision Livestock Farming on a Productivity Index at Farm Level. Some Evidences from a Dairy Farms' Sample of Lombardy. Animals 2020, Vol. 10, Page 1781 10. Multidisciplinary Digital Publishing Institute. https://doi.org/10.3390/ANI10101781.
Çetin, Bahattin, Arzu Akpinar, and Damla Özsayin. 2016. Use of ICT as a Critical Marketing Success Factor in Turkish Agri-Food SMEs: Outlook on Agriculture 33. SAGE PublicationsSage UK: London, England: 215–218. https://doi.org/10.5367/0000000042530222.
Chikuni, Thokozani, and Fredy T.M. Kilima. 2019. Smallholder farmers' market participation and mobile phone-based market information services in Lilongwe, Malawi. The Electronic Journal of Information Systems in Developing Countries 85. John Wiley & Sons, Ltd: e12097. https://doi.org/10.1002/ISD2.12097.
D'Antoni, Jeremy M., Ashok K. Mishra, and Hyunjeong Joo. 2012. Farmers' perception of precision technology: The case of autosteer adoption by cotton farmers. Computers and Electronics in Agriculture 87. Elsevier: 121–128. https://doi.org/10.1016/J.COMPAG.2012.05.017.
Daum, Thomas, Roberto Villalba, Oluwakayode Anidi, Sharon Masakhwe Mayienga, Saurabh Gupta, and Regina Birner. 2021. Uber for tractors? Opportunities and challenges of digital tools for tractor hire in India and Nigeria. World Development 144. Pergamon. https://doi.org/10.1016/J.WORLDDEV.2021.105480.
Dissanayeke, Uvasara, and W. A.D.P. Wanigasundera. 2014. Mobile Based Information Communication Interactions among Major Agriculture Stakeholders: Sri Lankan Experience. The Electronic Journal of Information Systems in Developing Countries 60. John Wiley & Sons, Ltd: 1–12. https://doi.org/10.1002/J.1681-4835.2014.TB00422.X.
Drewry, Jessica L., John M. Shutske, David Trechter, Brian D. Luck, and Lynn Pitman. 2019. Assessment of digital technology adoption and access barriers among crop, dairy and livestock producers in Wisconsin. Computers and Electronics in Agriculture 165. Elsevier. https://doi.org/10.1016/J.COMPAG.2019.104960.
Groher, T., K. Heitkämper, and C. Umstätter. 2020. Digital technology adoption in livestock production with a special focus on ruminant farming. Animal 14. Elsevier: 2404–2413. https://doi.org/10.1017/S1751731120001391.
Hartmann, Gideon, Gilbert Nduru, and Peter Dannenberg. 2020. Digital connectivity at the upstream end of value chains: A dynamic perspective on smartphone adoption amongst horticultural smallholders in Kenya: Competition and Change 25. SAGE PublicationsSage UK: London, England: 167–189. https://doi.org/10.1177/1024529420914483.
Hay, Rachel, and Philip Pearce. 2014. Technology adoption by rural women in Queensland, Australia: Women driving technology from the homestead for the paddock. Journal of Rural Studies 36. Pergamon: 318–327. https://doi.org/10.1016/J.JRURSTUD.2014.10.002.
Hoang, Hung Gia. 2020. Determinants of the adoption of mobile phones for fruit marketing by Vietnamese farmers. World Development Perspectives 17. Elsevier: 1–8. https://doi.org/10.1016/J.WDP.2020.100178.
Kante, Macire, Robert Oboko, and Christopher Chepken. 2017. Influence of Perception and Quality of ICT-Based Agricultural Input Information on Use of ICTs by Farmers in Developing Countries: Case of Sikasso in Mali. The Electronic Journal of Information Systems in Developing Countries 83. John Wiley & Sons, Ltd: 1–21. https://doi.org/10.1002/J.1681-4835.2017.TB00617.X.
Kante, Macire, Robert Oboko, and Christopher Chepken. 2019. An ICT model for increased adoption of farm input information in developing countries: A case in Sikasso, Mali. Information Processing in Agriculture 6. Elsevier: 26–46. https://doi.org/10.1016/J.INPA.2018.09.002.
Karanja, Lucy, Stephanie Gakuo, Monica Kansiime, Dannie Romney, Henry Mibei, James Watiti, Leonard Sabula, and Daniel Karanja. 2020. Impacts and challenges of ICT based scale-up campaigns: Lessons learnt from the use of SMS to support maize farmers in the UPTAKE project, Tanzania. Data Science Journal 19. Ubiquity Press: 1–8. https://doi.org/10.5334/DSJ-2020-007/METRICS/.
Kernecker, Maria, Andrea Knierim, Angelika Wurbs, Teresa Kraus, and Friederike Borges. 2020. Experience versus expectation: farmers' perceptions of smart farming technologies for cropping systems across Europe. Precision Agriculture 21. Springer: 34–50. https://doi.org/10.1007/S11119-019-09651-Z/TABLES/3.
Khan, Nasir Abbas, Gao Qijie, Selamawit Fantahun Sertse, Md Nur Nabi, and Palwasha Khan. 2019. Farmers' use of mobile phone-based farm advisory services in Punjab, Pakistan: Information Development 36. SAGE PublicationsSage UK: London, England: 390–402. https://doi.org/10.1177/0266666919864126.
Krell, N. T., S. A. Giroux, Z. Guido, C. Hannah, S. E. Lopus, K. K. Caylor, and T. P. Evans. 2021. Smallholder farmers' use of mobile phone services in central Kenya. Climate and Development 13. Taylor and Francis Ltd.: 215–227. https://doi.org/10.1080/17565529.2020.1748847/SUPPL_FILE/TCLD_A_1748847_SM2839.DOCX.
Larson, James A., Roland K. Roberts, Burton C. English, Sherry L. Larkin, Michele C. Marra, Steven W. Martin, Kenneth W. Paxton, and Jeanne M. Reeves. 2008. Factors affecting farmer adoption of remotely sensed imagery for precision management in cotton production. Precision Agriculture 9. Springer: 195–208. https://doi.org/10.1007/S11119-008-9065-1/TABLES/3.
Lencsés, Eniko, István Takács, and Katalin Takács-György. 2014. Farmers' Perception of Precision Farming Technology among Hungarian Farmers. Sustainability 6. Multidisciplinary Digital Publishing Institute: 8452–8465. https://doi.org/10.3390/SU6128452.
Leng, Chenxin, Wanglin Ma, Jianjun Tang, and Zhongkun Zhu. 2020. ICT adoption and income diversification among rural households in China. Applied Economics 52. Routledge: 3614–3628. https://doi.org/10.1080/00036846.2020.1715338.
López-Becerra, Erasmo I., Narciso Arcas-Lario, and Francisco Alcon. 2016. The websites adoption in the Spanish agrifood firms. Spanish Journal of Agricultural Research 14. Ministerio de Agricultura Pesca y Alimentacion. https://doi.org/10.5424/SJAR/2016144-10113.
McCampbell, Mariette, Julius Adewopo, Laurens Klerkx, and Cees Leeuwis. 2021. Are farmers ready to use phone-based digital tools for agronomic advice? Ex-ante user readiness assessment using the case of Rwandan banana farmers. The Journal of Agriculture Education and Extension. Routledge. https://doi.org/10.1080/1389224X.2021.1984955.
Michels, Marius, Vanessa Bonke, and Oliver Musshoff. 2020. Understanding the adoption of smartphone apps in crop protection. Precision Agriculture 21. Springer: 1209–1226. https://doi.org/10.1007/S11119-020-09715-5/TABLES/6.
Michels, Marius, Wilm Fecke, Jan Henning Feil, Oliver Musshoff, Frederike Lülfs-Baden, and Saskia Krone. 2020. “Anytime, anyplace, anywhere”—A sample selection model of mobile internet adoption in german agriculture. Agribusiness 36. John Wiley & Sons, Ltd: 192–207. https://doi.org/10.1002/AGR.21635.
Michels, Marius, Wilm Fecke, Jan Henning Feil, Oliver Musshoff, Johanna Pigisch, and Saskia Krone. 2020. Smartphone adoption and use in agriculture: empirical evidence from Germany. Precision Agriculture 21. Springer: 403–425. https://doi.org/10.1007/S11119-019-09675-5/TABLES/4.
Mitchell, Sean, Alfons Weersink, and Bruce Erickson. 2018. Adoption of precision agriculture technologies in ontario crop production. Canadian Journal of Plant Science 98. Agricultural Institute of Canada: 1384–1388. https://doi.org/10.1139/CJPS-2017-0342/ASSET/IMAGES/LARGE/CJPS-2017-0342F1.JPEG.
Mwalupaso, Gershom Endelani, Shangao Wang, Zhangxing Xu, and Xu Tian. 2019. Towards Auspicious Agricultural Informatization—Implication of Farmers' Behavioral Intention Apropos of Mobile Phone Use in Agriculture. Sustainability  11. Multidisciplinary Digital Publishing Institute. https://doi.org/10.3390/SU11226282.
Okello, Dickson Otieno, Shiferaw Feleke, Edith Gathungu, George Owuor, and Oscar Ingasia Ayuya. 2020. Effect of ICT tools attributes in accessing technical, market and financial information among youth dairy agripreneurs in Tanzania. Cogent Food and Agriculture 6. Cogent. https://doi.org/10.1080/23311932.2020.1817287.
Ortiz-Crespo, Berta, Jonathan Steinke, Carlos F. Quirós, Jeske van de Gevel, Happy Daudi, Majuto Gaspar Mgimiloko, and Jacob van Etten. 2020. User-centred design of a digital advisory service: enhancing public agricultural extension for sustainable intensification in Tanzania. International Journal of Agriculture Sustainability 19. Taylor & Francis: 566–582. https://doi.org/10.1080/14735903.2020.1720474.
Owusu, Alex Barimah, Paul W.K. Yankson, and Stephen Frimpong. 2017. Smallholder farmers' knowledge of mobile telephone use: Gender perspectives and implications for agricultural market development: Progress in Development Studies 18. SAGE PublicationsSage India: New Delhi, India: 36–51. https://doi.org/10.1177/1464993417735389.
Paustian, Margit, and Ludwig Theuvsen. 2017. Adoption of precision agriculture technologies by German crop farmers. Precision Agriculture 18. Springer New York LLC: 701–716. https://doi.org/10.1007/S11119-016-9482-5/TABLES/3.
Pede, Valerien, Takashi Yamano, Prakashan Chellattanveettil, and Ishika Gupta. 2018. Receiving information about rice seeds on mobile phones in eastern India. Development in Practice 28. Routledge: 95–106. https://doi.org/10.1080/09614524.2018.1397105.
Pivoto, Dieisson, Bradford Barham, Paulo Dabdab Waquil, Cristian Rogério Foguesatto, Vitor Francisco Dalla Corte, Debin Zhang, and Edson Talamini. 2019. Factors influencing the adoption of smart farming by Brazilian grain farmers. International Food and Agribusiness Management Review 22.  Wageningen Academic Publishers : 571–588. https://doi.org/10.22434/IFAMR2018.0086.
Raheem, Dele. 2020. Digitalisation in a local food system: Emphasis on Finnish Lapland. Open Agriculture 5. De Gruyter Open Ltd: 496–508. https://doi.org/10.1515/OPAG-2020-0049/MACHINEREADABLECITATION/RIS.
Rajkhowa Id, Pallavi, and Matin Qaim Id. 2021. Personalized digital extension services and agricultural performance: Evidence from smallholder farmers in India. Edited by Bjorn Van Campenhout. PLOS ONE 16. Public Library of Science: e0259319. https://doi.org/10.1371/JOURNAL.PONE.0259319.
Schulz, Penelope, Julian Prior, Lewis Kahn, and Geoff Hinch. 2021. Exploring the role of smartphone apps for livestock farmers: data management, extension and informed decision making. The Journal of Agriculture Education and Extension. Routledge. https://doi.org/10.1080/1389224X.2021.1910524.
Sheng, Jie, and Qian Lu. 2020. The influence of information communication technology on farmers' sales channels in environmentally affected areas of China. Environmental Science and Pollution Research 27. Springer Science and Business Media Deutschland GmbH: 42513–42529. https://doi.org/10.1007/S11356-020-10203-6/TABLES/9.
Tamirat, Tseganesh Wubale, Søren Marcus Pedersen, and Kim Martin Lind. 2017. Farm and operator characteristics affecting adoption of precision agriculture in Denmark and Germany. Acta Agriculturae Scandinavica 68. Taylor & Francis: 349–357. https://doi.org/10.1080/09064710.2017.1402949.
Thar, So Pyay, Thiagarajah Ramilan, Robert J. Farquharson, Alexis Pang, and Deli Chen. 2021. An empirical analysis of the use of agricultural mobile applications among smallholder farmers in Myanmar. The Electronic Journal of Information Systems in Developing Countries 87. John Wiley & Sons, Ltd: e12159. https://doi.org/10.1002/ISD2.12159.
Vecchio, Yari, Giulio Paolo Agnusdei, Pier Paolo Miglietta, and Fabian Capitanio. 2020. Adoption of Precision Farming Tools: The Case of Italian Farmers. International Journal of Environmental Research and Public Health 2020, Vol. 17, Page 869 17. Multidisciplinary Digital Publishing Institute. https://doi.org/10.3390/IJERPH17030869.
Voss, Rachel C., Tony Jansen, Bacary Mané, Carol Shennan, Rachel C. Voss, Tony Jansen, Bacary Mané, and Carol Shennan. 2021. Encouraging technology adoption using ICTs and farm trials in Senegal: Lessons for gender equity and scaled impact. World Development 146. Elsevier. https://doi.org/10.1016/J.WORLDDEV.2021.105620.
Walton, Jonathan C., Dayton M. Lambert, Roland K. Roberts, James A. Larson, Burton C. English, Sherry L. Larkin, Steven W. Martin, Michele C. Marra, Kenneth W. Paxton, and Jeanne M. Reeves. 2008. Adoption and Abandonment of Precision Soil Sampling in Cotton Production. Journal of Agricultural and Resource Economics 33: 428–448. https://doi.org/10.22004/AG.ECON.46556.
Yoon, Cheolho, Dongsup Lim, and Changhee Park. 2020. Factors affecting adoption of smart farms: The case of Korea. Computers in Human Behavior 108. Pergamon. https://doi.org/10.1016/J.CHB.2020.106309.
Yu, Lili, Duanyang Zhao, Zihao Xue, and Yang Gao. 2020. Research on the use of digital finance and the adoption of green control techniques by family farms in China. Technology in Society 62. Pergamon: 101323. https://doi.org/10.1016/J.TECHSOC.2020.101323.
Zheng, Hongyun, and Wanglin Ma. 2021. Smartphone-based information acquisition and wheat farm performance: insights from a doubly robust IPWRA estimator. Electronic Commerce Research. Springer: 1–26. https://doi.org/10.1007/S10660-021-09481-0/TABLES/10.


Supplemental Figure S3:  Sub-group analysis


Supplemental Figure S4: Funnel plot by Group


Supplemental S5: Egger's test

Meta bias, egger
Effect-size label:  adoption
Effect size:  _ES
Std. Err.:  _seES
Regression-based Egger test for small-study effects
Random-effects model
Method: REML
H0: beta1 = 0; no small-study effects
beta1 =      3.83
SE of beta1 =     1.191
z =      3.22
Prob > |z| =    0.0013

Nonparametric trim-and-fill analysis of publication bias
Run estimator, imputing on the left
Iteration                            Number of studies =     94
Model: Random-effects                       Observed =     68
 Method: REML                                  Imputed =     26
Pooling
Model: Random-effects
Method: REML
 Studies 	 Adoption	 [95%Conf.	 Interval]	
Observed 	    0.395	    0.332	    0.458	
Observed + Imputed 	    0.218	    0.141	    0.295	


Supplemental Figure S6: Effect size of socioeconomics
-	Effect size or ''Age''


-	Effect size of Gender 


-	Effect size of education


-	Effect size of farm size

-	Effect size of Household income


Supplemental S7 
24a- The review did not include any Randomized Control Trial or clinical studies; therefore, we did not register the protocol. 
24b- The review protocol is available with the corresponding author and can be provided to anyone interested in.
24c- Protocol was not registered.
